# Lifetime physical activity and breast cancer risk in the Shanghai Breast Cancer Study

**DOI:** 10.1054/bjoc.2000.1671

**Published:** 2001-04

**Authors:** C E Matthews, X-O Shu, F Jin, Q Dai, J R Hebert, Z-X Ruan, Y-T Gao, W Zheng

**Affiliations:** Department of Epidemiology and Biostatistics, University of South Carolina School of Public Health and the South Carolina Cancer Center, Columbia, SC, 29208; Department of Pediatrics and Epidemiology, University of South Carolina, Columbia, SC, 29203; Department of Epidemiology, Shanghai Cancer Institute, Shanghai, 200032, People's Republic of China; Vanderbilt Center for Health Services Research and the Vanderbilt-Ingram Cancer Center, Vanderbilt University, Nashville, TN, 37232

**Keywords:** exercise, epidemiology, breast cancer, prevention

## Abstract

Overall physical activity in adolescence and adulthood, and changes in activity over the lifespan were analysed by in-person interviews among 1459 women newly diagnosed with breast cancer and 1556 age-matched controls in urban Shanghai. Physical activity from exercise and sports, household, and transportation (walking and cycling) was assessed in adolescence (13–19 y) and adulthood (last 10 y), as was lifetime occupational activity. Logistic regression was used to estimate odds ratios (OR) and 95% confidence limits (OR (95% CL)) while controlling for confounders. Risk was reduced for exercise only in adolescence (OR = 0.84 (0.70–1.00)); exercise only in adulthood (OR = 0.68 (0.53–0.88)), and was further reduced for exercise in both adolescence and adulthood (OR = 0.47 (0.36–0.62)). Graded reductions in risk were noted with increasing years of exercise participation (OR _1–5 yrs_= 0.81 (0.67–0.94); OR _6–10 yrs_= 0.74 (0.59–0.93); OR _11–15 yrs_= 0.55 (0.38–0.79); OR _16 + yrs_= 0.40 (0.27–0.60);*P*_trend,_< 0.01). Lifetime occupational activity also was inversely related to risk (*P*_trend_< 0.01). These findings demonstrate that consistently high activity levels throughout life reduce breast cancer risk. Furthermore, they suggest that women may reduce their risk by increasing their activity levels in adulthood. © 2001 Cancer Research Campaignhttp://www.bjcancer.com


*British Journal of Cancer* (2001) **84**(7), 994–1001
© 2001 Cancer Research Campaign
doi: 10.1054/ bjoc.2001.1671, available online at http://www.idealibrary.com on IDEAL®

http://www.bjcancer.com

### Lifetime physical activity and breast cancer risk in the Shanghai Breast Cancer Study

**CE Matthews[1], X-O Shu[1,2,4], F Jin[3], Q Dai[1,3,4], JR Hebert[1], Z-X Ruan[3], Y-T Gao[3] and W Zheng[1,4]**

[1]Department of Epidemiology and Biostatistics, University of South Carolina School of Public Health and the South Carolina Cancer Center, Columbia, SC 29208; [2]Department of Pediatrics and Epidemiology, University of South Carolina, Columbia, SC 29203; [3]Department of Epidemiology, Shanghai Cancer Institute, Shanghai, People's Republic of China 200032; [4]Vanderbilt Center for Health Services Research and the Vanderbilt-Ingram Cancer Center, Vanderbilt University, Nashville, TN 37232

**Summary** Overall physical activity in adolescence and adulthood, and changes in activity over the lifespan were analysed by in-person interviews among 1459 women newly diagnosed with breast cancer and 1556 age-matched controls in urban Shanghai. Physical activity from exercise and sports, household, and transportation (walking and cycling) was assessed in adolescence (13–19 y) and adulthood (last 10 y), as was lifetime occupational activity. Logistic regression was used to estimate odds ratios (OR) and 95% confidence limits (OR (95% CL)) while controlling for confounders. Risk was reduced for exercise only in adolescence (OR = 0.84 (0.70–1.00)); exercise only in adulthood (OR = 0.68 (0.53–0.88)), and was further reduced for exercise in both adolescence and adulthood (OR = 0.47 (0.36–0.62)). Graded reductions in risk were noted with increasing years of exercise participation ($OR_{1–5\ yrs}$ = 0.81 (0.67–0.94); $OR_{6–10\ yrs}$ = 0.74 (0.59–0.93); $OR_{11–15\ yrs}$ = 0.55 (0.38–0.79); $OR_{16+\ yrs}$ = 0.40 (0.27–0.60); $P_{trend,}$ < 0.01). Lifetime occupational activity also was inversely related to risk ($P_{trend}$ < 0.01). These findings demonstrate that consistently high activity levels throughout life reduce breast cancer risk. Furthermore, they suggest that women may reduce their risk by increasing their activity levels in adulthood. © 2001 Cancer Research Campaign http://www.bjcancer.com

**Keywords**: exercise; epidemiology; breast cancer; prevention

Frisch and colleagues (1985) reported that women who did not participate in collegiate athletics were at 1.86 times greater risk of breast cancer relative to collegiate athletes, 82% of whom were physically active in adolescence and 74% of whom maintained high physical activity levels into adulthood. Most subsequent studies have provided evidence for a reduced risk of breast cancer among women with high levels of physical activity (Friedenreich et al, 1998b). Inconsistencies in previous studies may be partly due to inadequate measures of long-term physical activity. Two recent prospective studies employing multiple measures of physical activity in adulthood have reported 20 to 50% reductions in risk (Thune et al, 1997; Rockhill et al, 1999). While case–control studies may be more susceptible to recall and selection biases, they offer the opportunity, unavailable in most prospective studies, to obtain more comprehensive assessments of lifetime physical activity.

As few recent studies have done so, we have examined the effects of lifetime occupational physical activity, and the independent effects on breast cancer risk of exercise in adolescence and adulthood, both separately and together as well as changes between adolescence and adulthood.

### MATERIALS AND METHODS

Study design and participant recruitment. Relevant bodies approved the methodology for the Shanghai Breast Cancer Study and a detailed description of the study has been published elsewhere (Gao et al, 2000). Briefly, this was a population-based case–control study designed to recruit all incident breast cancer cases aged 25 to 64 years among permanent residents of urban Shanghai between August 1996 and March 1998. All study participants had no prior history of cancer and were alive at the time of interview. Through a rapid case-ascertainment system, supplemented by the population-based Shanghai Cancer Registry, 1602 eligible breast cancer cases were identified during the study period, and in-person interviews were completed for 1459 (91.1%) of them. The major reasons for non-participation were refusal (109 cases, 6.8%), death prior to interview (17 cases, 1.1%), and inability to locate (17 cases, 1.1%). Two senior pathologists confirmed the diagnoses.

Controls were randomly selected from permanent female residents of urban Shanghai and frequency-matched to cases by age. The number of controls in each age-specific stratum was determined in advance using the age distribution of the incident breast cancer cases reported to the Shanghai Cancer Registry from 1990 to 1993. This registry, which keeps cards for all permanent residents of urban Shanghai, was used to select controls. For each age-predetermined control, a registry card for a potential control in the same 5-year age group was randomly selected. In-person interviews were completed for 1556 (90.3%) of the 1724 eligible controls identified. Only 166 (9.6%) controls refused participation and 2 (0.1%) controls were excluded because they were deceased.

### Measurements

Trained interviewers used a structured questionnaire in hospitals (cases) or homes (cases and controls) to collect details of

*Received 16 October 2000*
*Revised 5 January 2001*
*Accepted 9 January 2001*

*Correspondence to:* CE Matthews demography, occupational and non-occupational physical activity, menstrual and reproductive history, hormone use, diet, disease history, tobacco and alcohol use, weight, and family history of cancer. Participants were also weighed, waist and hip circumferences and sitting and standing heights were measured and waist:hip ratio and body mass index (BMI, kg/m$^2$) calculated.

Details were also obtained about lifetime occupational activity, while other activities (viz. exercise and sports, household, and transportation activities (walking, cycling)) were assessed for the adolescent period (13–19 y) and in the 10 years prior to entering the study (adulthood). Women reported each occupation they held for at least 3 years during their lifetime, and the calendar year in which they started and ended each job. Because of the general stability of occupations in China, this approach captures the majority of jobs held in a lifetime and their duration; these were classified into high, medium, or low levels of estimated sitting time and activity level using job codes from the more than 200 different occupations reported. Examples of those categorized as high sitting time and low-activity categories were clerks and accountants, while those in the low sitting time and higher-activity categories included sales clerks, cooks and machine operators. In addition, for each occupation, participants reported the average time spent in ‘standing or walking’ (h d$^{-1}$), and classified each into one of four activity categories (i.e., heavy, medium, light or non-physical work). Summary measures were calculated by multiplying the years spent in each occupation by the specific activity variable, and then summing the result over all jobs.

Women could report up to five exercise or sport activities during each life-period, as well as the average duration (min d$^{-1}$) and the length of participation (y) in each activity. Only 13 women (0.4%) reported five exercise activities in adolescence, and no women reported five activities in adulthood. Among women reporting exercise or sports participation, a three-category question regarding sweating was also completed. Participants also were asked, for each life-period, to categorize their exercise and sports activity, relative to their peers, into one of five categories. Since there is little inter-individual variation in adolescent sports participation in physical education classes, details of sports at school were not collected. Quantitative estimates of energy expenditure in sports and exercise were calculated as MET-hours day$^{-1}$ year$^{-1}$ (MET-h d$^{-1}$ y$^{-1}$) using standardized metabolic equivalent (MET) values (Ainsworth et al, 2000), activity duration (h d$^{-1}$), and the length of activity participation (y). Additionally, the total number of exercise-years accumulated in adolescence and adulthood was calculated by summing the total number of years reported in all exercise and sport activities in each life-period (e.g., 3 sports played for 4 y each = 12 exercise-years). Women were asked to report the number of hours per week they spent in housework and the duration (min d$^{-1}$) spent walking and cycling each day both in adolescence and adulthood, but the lengths of activity participation in each life-period were not obtained.

### Statistical analysis

Unconditional logistic regression models were used to obtain maximum likelihood estimates of the odds ratios and their 95% confidence limits (95% CL) (Hosmer and Lemeshow, 1989). Results were presented for models with minimal adjustment (age (y)) and in adjusted models. A number of risk factors, previously noted to be independently associated with breast cancer risk in this population (Gao et al, 2000), were controlled in fully adjusted models, including: breast cancer in first-degree relative (0,1), history of breast fibroadenoma (0,1), age at menarche (y), age at first live birth (y), menopause (0,1) and age at menopause (<45 y, 45–50 y, ≥50 y). Fully adjusted models also controlled for age (y), education (no formal education, elementary school, middle and high school, college and above) and household income (<4000, 4000–5999, 6000–7999, 8000–8999, 9000+ Yuan). Linear trends in the risk estimates were tested statistically by entering continuous parameters in the models rather than the dummy coded categorical physical activity exposure variables employed to estimate odds ratios. Classifications of activity by quartile (for exercisers) or quintile (occupational activity) were made using the exposure distributions of the control subjects. For evaluation of cumulative exposure (i.e., exercise-years) we used categories of roughly 5-year exposure increments (e.g., 1–5 y, 6–10 y, etc). Since physical activity energy expenditure is the most modifiable component of the energy balance equation, we considered BMI to be in the causal pathway between physical activity and breast cancer risk, and therefore did not control for BMI in our models.

### RESULTS

The descriptive characteristics for cases and controls in the Shanghai Breast Cancer Study are presented in Table 1. Briefly, cases were slightly older, had higher waist:hip ratios and BMI levels, earlier age at menarche, later age at first live birth and menopause, and proportionally more breast fibroadenomas and first-degree family history of breast cancer. Cases also tended to have more college education and higher household incomes.

Inverse associations for all exercise measures (MET-h d$^{-1}$ y$^{-1}$, self-comparison, sweating) in adolescence (13–19 y) and adulthood (last 10 years) are reported in Table 2. Adjustment for a number of important breast cancer risk factors resulted in only minor alterations in the risk estimates. In fully adjusted 2 × 2 analyses (i.e., exercise = Yes or No), the adult risk estimate was OR = 0.61 (0.50–0.73) and the adolescent value was OR = 0.77 (0.66–0.90).

Women reporting a lifetime average of more than one hour per day of standing or walking at work had a 20–40% reduced breast cancer risk (Table 3). Self-ratings of occupational activity and estimated sitting time, based on job codes, were inversely associated with risk in age-, but not in fully adjusted models (Table 3). Findings for occupational activity based on job codes were null.

Examination of the effect of changes in exercise behaviour between adolescence and adulthood among all women revealed that women reporting exercise in both life-periods had breast cancer risk reductions of 53% (i.e., Yes/Yes), relative to women who did not exercise in either life-period, after adjusting for potential confounding factors (Table 4). Women who initiated exercise in adulthood had risk reductions of 32%, while women who exercised only in adolescence had reductions of 16% (Table 4). These findings were not materially altered after controlling for occupational activity (i.e., standing or walking (h d$^{-1}$). Only 21% of the population reported exercise in adulthood, and only half of this group of exercisers reported exercising in both life-periods (i.e., 10% of the overall population).

Both pre- and post-menopausal women who were consistent exercisers in adolescence and adulthood experienced the greatest reductions in risk (i.e., Yes/Yes: $OR_{pre-}$ = 0.57 (0.40–0.82); $OR_{post-}$ = 0.36 (0.24–0.55). Pre- and post-menopausal women who

© 2001 Cancer Research Campaign

**Table 1** Descriptive characteristics of women in the Shanghai Breast Cancer Study (1996–1998), by disease status. Values are mean (SD) or the percent of total reporting (%)

| | Case (*n* = 1459) | Control (*n* = 1556) | *P* * |
|---|---|---|---|
| Age (years) | 47.9 (8.0) | 47.2 (8.8) | 0.03 |
| Height (cm) | 158.9 (5.1) | 158.5 (5.4) | 0.07 |
| BMI (kg/m²) | 23.5 (3.4) | 23.1 (3.4) | < 0.01 |
| Waist: hip | 0.81 (0.06) | 0.80 (0.06) | < 0.01 |
| Education (%) | | | |
| No formal education | 3.6 | 5.5 | 0.01 |
| Elementary school | 8.5 | 8.4 | |
| Middle & high school | 74.3 | 75.5 | |
| College and above | 13.6 | 10.7 | |
| Household Income (Yuan) (%) | | | |
| < 4000 | 19.8 | 18.3 | 0.05 |
| 4000–5999 | 31.7 | 31.9 | |
| 6000–7999 | 13.3 | 14.0 | |
| 8000–8999 | 20.2 | 23.5 | |
| 9000 + | 15.2 | 12.4 | |
| Breast cancer in first-degree relative (%) | 3.7 | 2.4 | 0.05 |
| Ever had breast fibroadenoma (%) | 9.6 | 5.0 | < 0.01 |
| Age at menarche (y) | 14.5 (1.6) | 14.7 (1.7) | < 0.01 |
| Parity (%) | 95.7 | 97.1 | 0.04 |
| Number of full-term pregnancies | 1.5 (0.9) | 1.5 (0.9) | 0.63 |
| Age at first live birth (y) | 26.7 (4.2) | 26.2 (3.9) | < 0.01 |
| Post-menopausal (%) | 34.5 | 36.2 | 0.32 |
| Age at menopause (y) | 48.1 (4.6) | 47.5 (4.9) | 0.02 |

*P* *, test differences in mean or frequency distributions by disease status.

exercised only in adolescence appeared to have modest reductions in risk (i.e., Yes/No: $OR_{pre-}$ = 0.85 (0.69–1.05)); $OR_{post-}$ = 0.81 (0.57–1.14)), although the confidence limits included the null value. The effect of initiating exercise in adulthood was confined to postmenopausal women (i.e., No/Yes–$OR_{post-}$ = 0.55 (0.40–0.77)) (Table 4). Evaluation of the cumulative exposure to exercise participation among ever exercisers, in adolescence or adulthood, revealed that women with more than 10 exercise-years (i.e., sum of years in all exercises combined) experienced a 55–76% reduction in risk relative to non-exercisers. The risk reduction with increasing length of exercise participation was graded and appeared to be stronger in post-menopausal women.

There was little evidence, aside from a weak non-significant effect for household activity in adolescence, for a beneficial effect of household activity, overall walking, or cycling in adolescence or adulthood (Table 5), possibly due to limitations in our activity assessment.

The effect of lifetime patterns of exercise across levels of occupational standing or walking, with non-exercisers in both life-periods (i.e., No/No) and the lower quintile of occupational activity as referent, are presented in Table 6. There appeared to be no consistent effect modification of exercise in each life-period (i.e., Yes/Yes) across occupational activity levels (Table 6). In general, the beneficial effects of exercise only in adolescence or adulthood were somewhat stronger among more occupationally active women (i.e., >2.4 h $d^{-1}$) (Table 6).

We evaluated our primary exercise and occupational activity findings among overweight women (BMI >25 kg/$m^{-2}$). Among overweight pre-menopausal women, exercise in both adolescence and adulthood was associated with reduced risk (OR = 0.34 (0.15–0.78)). Among overweight post-menopausal women, exercise in both adolescence and adulthood was associated with reduced risk (OR = 0.40 (0.15–0.78)), as was initiating exercise in adulthood (OR = 0.46 (0.28–0.78)). Though obesity (BMI 30+ kg $m^{-2}$) was rare in this population (4%), exercise in either adolescence or adulthood was associated with reduced risk in pre- (OR = 0.49 (0.11–2.11)) and post-menopausal (OR = 0.31 (0.12–0.82)) obese women. No effect modification by BMI level was noted in the inverse relationship between lifetime averages of occupational standing or walking and breast cancer.

### DISCUSSION

The present findings add to the evidence of an inverse relationship between lifetime physical activity and risk of breast cancer. Pre-and post-menopausal women who exercised in both adolescence and adulthood had reductions in risk than exercise throughout life of 43% and 64%, respectively. Importantly, initiating exercise in adulthood was associated with a 32% reduction in risk, while exercise only in adolescence reduced risk by 16%. That exercise only in adolescence may be less strongly associated with risk than exercise throughout life was supported by a clear dose–response between years of exercise participation and reduced risk of breast cancer in both pre- and post-menopausal women. Our findings were not substantially modified among overweight women (BMI > 25 kg/$m^2$), and little changed after controlling for occupational activity (i.e., standing or walking (h $d^{-1}$)) and several established breast cancer risk factors. They also are consistent with certain case–control studies (Bemstein et al, 1994; Carpenter et al, 1999; Friedenreich et al, 2001) and two cohort studies using serial measurements of physical activity (Thune et al, 1997; Rockhill et al, 1999). Serial assessments would be more likely to identify women who maintained habitually high activity levels throughout adulthood.

Studies examining physical activity at only a single point in time have been difficult to interpret due to variations in activity

 *© 2001 Cancer Research Campaign*

**Table 2** Odds ratios (OR) and 95% confidence limits (95% CL) for breast cancer associated with adolescent and adult exercise from age- and fully-adjusted models, the Shanghai Breast Cancer Study (1996–1998)

| | Age-adjusted | | | Fully-adjusted[1] | |
|---|---|---|---|---|---|
| | Case/Control[2] | OR | (95% CL) | OR | (95% CL) |
| *Adolescent (13–19 yrs)* | | | | | |
| Exercise (MET h $d^{-1}$ $y^{-1}$) | | | | | |
| No exercise | 960/941 | 1.00 | – | 1.00 | – |
| 0.01–0.75 | 132/150 | 0.88 | (0.68–0.75) | 0.82 | (0.63–1.07) |
| 0.76–1.93 | 146/162 | 0.89 | (0.70–1.13) | 0.90 | (0.70–1.16) |
| 1.94–4.29 | 135/152 | 0.88 | (0.69–1.14) | 0.82 | (0.63–1.06) |
| 4.30 + | 86/151 | 0.57 | (0.43–1.13) | 0.52 | (0.39–0.70) |
| *P* for trend | | < 0.01 | | <0.01 | |
| Exercise (self-comparison) | | | | | |
| No exercise | 960/935 | 1.00 | – | 1.00 | – |
| Less than average | 17/24 | 0.69 | (0.37–1.29) | 0.67 | (0.34–1.30) |
| A little less than average | 44/45 | 0.97 | (0.63–1.48) | 0.91 | (0.58–1.43) |
| About average | 226/282 | 0.80 | (0.65–0.97) | 0.75 | (0.61–0.92) |
| A little more than average | 158/196 | 0.80 | (0.63–1.00) | 0.77 | (0.60–0.97) |
| More than average | 53/67 | 0.78 | (0.54–1.12) | 0.74 | (0.51–1.09) |
| *P* for trend | | 0.01 | | < 0.01 | |
| Exercise (sweating) | | | | | |
| No exercise | 960/935 | 1.00 | – | 1.00 | – |
| Normally did not sweat | 203/252 | 0.83 | (0.65–1.08) | 0.80 | (0.61–1.04) |
| Sweated most of the time | 168/210 | 0.79 | (0.63–0.99) | 0.73 | (0.58–0.93) |
| Sweated every time | 127/151 | 0.80 | (0.65–0.98) | 0.77 | (0.62–0.95) |
| *P* for trend | | 0.02 | | < 0.01 | |
| *Adult (last 10 yrs)* | | | | | |
| Exercise (MET-h $d^{-1}$ $y^{-1}$) | | | | | |
| No exercise | 1193/1165 | 1.00 | – | 1.00 | – |
| 0.01–0.35 | 49/83 | 0.56 | (0.39–0.80) | 0.54 | (0.37–0.79) |
| 0.36–0.88 | 101/112 | 0.80 | (0.60–1.07) | 0.78 | (0.58–1.05) |
| 0.89–1.91 | 70/96 | 0.66 | (0.48–0.91) | 0.66 | (0.47–0.92) |
| 1.92 + | 46/97 | 0.40 | (0.28–0.58) | 0.40 | (0.27–0.59) |
| *P* for trend | | < 0.01 | | < 0.01 | |
| Exercise (self-comparison) | | | | | |
| No exercise | 1185/1163 | 1.00 | – | 1.00 | – |
| Less than average | 19/16 | 1.04 | (0.53–2.04) | 1.17 | (0.58–2.36) |
| A little less than average | 32/46 | 0.62 | (0.39–0.99) | 0.60 | (0.37–0.97) |
| About average | 138/221 | 0.55 | (0.44–0.70) | 0.55 | (0.43–0.71) |
| A little more than average | 74/82 | 0.86 | (0.62–1.19) | 0.84 | (0.60–1.20) |
| More than average | 11/26 | 0.40 | (0.20–0.82) | 0.35 | (0.16–0.76) |
| *P* for trend | | < 0.01 | | < 0.01 | |
| Exercise (sweating) | | | | | |
| No exercise | 1184/1163 | 1.00 | – | 1.00 | – |
| Normally did not sweat | 117/154 | 0.66 | (0.51–0.87) | 0.68 | (0.52–0.89) |
| Sweated most of the time | 75/118 | 0.58 | (0.42–0.78) | 0.56 | (0.41–0.77) |
| Sweated every time | 72/118 | 0.57 | (0.42–0.77) | 0.56 | (0.41–0.78) |
| *P* for trend | | < 0.01 | | < 0.01 | |

[1]Adjusted for age, education, household income, first-degree family history of breast cancer, history of breast fibroadenoma, age at menarche, age at first live birth, and age at menopause. [2]Numbers of cases and controls vary due to non-reporting on some variables.

patterns across the lifespan. For example, an early study (Frisch et al, 1985) reported that 82% of college athletes played sports in high school and 74% continued to exercise into adulthood (Frisch et al, 1985; Wyshak and Frisch, 2000). In contrast, in the non-athletic comparison group, only 25% of the women participated in high school athletics, and only 57 to 63% reported exercising later in life (Frisch et al, 1985; Wyshak and Frisch, 2000). Thus, classification of activity status at only one point in life, in this cohort at least, appears to have selected a group of predominantly lifelong exercisers. As such, the reported risk reductions of 40 to 50% may overestimate the apparent effect of exercise at a single point early in life (Frisch et al, 1985; Wyshak and Frisch, 2000).

Only one other published investigation has examined the independent effects of physical activity only in adolescence and only in adulthood as well as changes in activity over time. Modest reductions in risk (OR = 0.83 (0.60–1.16)) for only adolescent exercise among women aged 20–54 y (90% pre-menopausal) have been reported (Verloop et al, 2000); while those who initiated exercise in adulthood had a more pronounced risk reduction (OR = 0.65 (0.47–0.89)). Our data are remarkably consistent with this report and extend this finding to post-menopausal women. Confirmation of the finding that initiating exercise participation in adulthood, regardless of prior exercise patterns, can reduce risk is of great public health importance.

High levels of physical activity prior to menopause would be expected to minimize exposure to ovarian hormones by potentially delaying menarche (Merzenich et al, 1993), reducing the number of ovulatory menstrual cycles (Bernstein et al, 1987; De Souza

© 2001 Cancer Research Campaign 

**Table 3** Odds ratios (OR) and 95% confidence limits (95% CL) for breast cancer associated with lifetime occupational activity measures from age- and fully-adjusted models, the Shanghai Breast Cancer Study (1996–1998)

| | Age-adjusted | | | Fully-adjusted[1] | |
|---|---|---|---|---|---|
| | Case/Control[2] | OR | (95% CL) | OR | (95% CL) |
| *Self-reported activity* | | | | | |
| Standing or walking ($hd^{-1}$) | | | | | |
| 0.0–0.9 | 246/345 | 1.00 | – | 1.00 | – |
| 1.0–2.3 | 305/319 | 0.86 | (0.69–1.07) | 0.81 | (0.64–1.01) |
| 2.4–3.9 | 212/264 | 0.67 | (0.53–0.85) | 0.62 | (0.48–0.79) |
| 4.0–5.9 | 316/306 | 0.80 | (0.64–0.99) | 0.79 | (0.63–0.99) |
| 6.0 + | 361/300 | 0.59 | (0.47–0.74) | 0.61 | (0.48–0.77) |
| *P* for trend | | <0.01 | | <0.01 | |
| Activity self-rating[3] | | | | | |
| Q1 (low) | 280/336 | 1.00 | – | 1.00 | – |
| Q2 | 272/296 | 1.06 | (0.84–1.33) | 1.12 | (0.88–1.43) |
| Q3 | 259/310 | 0.81 | (0.64–1.03) | 0.83 | (0.65–1.07) |
| Q4 | 348/311 | 0.87 | (0.69–1.11) | 0.94 | (0.73–1.20) |
| Q5 (high) | 299/307 | 0.76 | (0.60–0.97) | 0.87 | (0.67–1.12) |
| | | <0.01 | | 0.09 | |
| *Job-code classifications*[3] | | | | | |
| Sitting time | | | | | |
| Q5 (longer) | 373/353 | 1.00 | – | 1.00 | – |
| Q4 | 320/284 | 1.10 | (0.88–1.38) | 1.04 | (0.82–1.32) |
| Q3 | 264/306 | 0.84 | (0.67–1.06) | 0.86 | (0.67–1.10) |
| Q2 | 278/332 | 0.82 | (0.66–1.03) | 0.89 | (0.69–1.14) |
| Q1 (shorter) | 224/281 | 0.81 | (0.63–1.04) | 0.90 | (0.68–1.20) |
| *P* for trend | | 0.01 | | 0.23 | |
| Activity level[3] | | | | | |
| Q1 (low) | 232/274 | 1.00 | – | 1.00 | – |
| Q2 | 325/334 | 1.14 | (0.91–1.43) | 1.00 | (0.79–1.26) |
| Q3 | 295/301 | 1.13 | (0.90–1.42) | 1.01 | (0.79–1.29) |
| Q4 | 325/334 | 1.15 | (0.92–1.45) | 1.00 | (0.79–1.27) |
| Q5 (high) | 282/313 | 1.06 | (0.82–1.37) | 0.92 | (0.70–1.22) |
| *P* for trend | | 0.62 | | 0.67 | |

[1]Adjusted for age, education, household income, first-degree family history of breast cancer, history of breast fibroadenoma, age at menarche, age at first live birth, and age at menopause. [2]Numbers of cases and controls vary due to non-reporting on some variables. [3]Q1–Q5 are quintiles of the physical activity exposure.

**Table 4** Odds ratios (OR) and 95% confidence limits (95% CL) for breast cancer associated with lifetime exercise patterns for age- and fully-adjusted models, the Shanghai Breast Cancer Study (1996–1998)

| | All women[1] | | | Pre-menopausal[2] | | | Post-menopausal[1] | | |
|---|---|---|---|---|---|---|---|---|---|
| | Case/Control[3] | OR | (95% CL) | Case/Control | OR | (95% CL) | Case/Control | OR | (95% CL) |
| Adolescent/Adult | | | | | | | | | |
| No/No | 806/745 | 1.00 | – | 534/517 | 1.00 | – | 270/226 | 1.00 | – |
| Yes/No | 387/420 | 0.84 | (0.70–1.00) | 290/320 | 0.85 | (0.69–1.05) | 95/98 | 0.81 | (0.57–1.14) |
| No/Yes | 154/194 | 0.68 | (0.53–0.88) | 60/54 | 0.95 | (0.63–1.41) | 93/140 | 0.55 | (0.40–0.77) |
| Yes/Yes | 112/194 | 0.47 | (0.36–0.62) | 68/99 | 0.57 | (0.40–0.82) | 43/95 | 0.36 | (0.24–0.55) |
| *P* for trend | | < 0.01 | | | <0.01 | | | <0.01 | |
| Ever exercise | | | | | | | | | |
| Zero exercise (y) | 805/745 | 1.00 | – | 534/517 | 1.00 | – | 269/226 | 1.00 | – |
| 1–5 exercise (y) | 352/385 | 0.81 | (0.67–0.97) | 229/239 | 0.88 | (0.70–1.11) | 121/146 | 0.67 | (0.49–0.92) |
| 6–10 exercise (y) | 195/244 | 0.74 | (0.59–0.93) | 125/147 | 0.82 | (0.62–1.08) | 69/95 | 0.63 | (0.43–0.92) |
| 11–15 exercise (y) | 59/85 | 0.55 | (0.38–0.79) | 35/45 | 0.63 | (0.39–1.03) | 24/40 | 0.45 | (0.25–0.80) |
| 16+ exercise (y) | 46/90 | 0.40 | (0.27–0.60) | 28/40 | 0.61 | (0.36–1.04) | 17/50 | 0.24 | (0.13–0.46) |
| *P* for trend | | <0.01 | | | <0.01 | | | <0.01 | |

[1]Adjusted for age, education, household income, first-degree family history of breast cancer, history of breast fibroadenoma, age at menarche, age at first live birth, and age at menopause. [2]Adjusted for age, education, household income, first-degree family history of breast cancer, history of breast fibroadenoma, age at menarche, age at first live birth. [3]Numbers of cases and controls vary due to non-reporting on some variables.

 © 2001 Cancer Research Campaign

**Table 5** Odds ratios (OR) and 95% confidence limits (95% CL) for breast cancer associated with household, walking, and cycling in adolescence and adulthood from age- and fully-adjusted models, the Shanghai Breast Cancer Study (1996–1998)

| | Age-adjusted | | | Fully-adjusted[1] | |
|---|---|---|---|---|---|
| | **Case/Control[2]** | **OR** | **(95% CL)** | **OR** | **(95% CL)** |
| *Adolescent (13–19 y)* | | | | | |
| Household activity ($d^{-1}$) | | | | | |
| Zero | 369/347 | 1.00 | – | 1.00 | – |
| 0.1–0.9 | 419/433 | 0.92 | (0.76–1.13) | 0.88 | (0.72–1.09) |
| 1.0–1.9 | 326/394 | 0.79 | (0.64–0.97) | 0.74 | (0.59–0.92) |
| 2.0–2.9 | 175/190 | 0.87 | (0.68–1.12) | 0.84 | (0.65–1.10) |
| ≥3.0 | 167/191 | 0.79 | (0.61–1.02) | 0.87 | (0.66–1.15) |
| *P* for trend | | 0.04 | | 0.13 | |
| Walking (min $d^{-1}$) | | | | | |
| 0–29 | 283/296 | 1.00 | – | 1.00 | – |
| 30–39 | 176/190 | 0.97 | (0.75–1.26) | 0.98 | (0.75–1.29) |
| 40–59 | 256/251 | 1.08 | (0.85–1.38) | 0.97 | (0.76–1.25) |
| 60–79 | 416/471 | 0.92 | (0.75–1.14) | 0.84 | (0.67–1.05) |
| ≥80 | 326/347 | 0.98 | (0.78–1.22) | 0.90 | (0.71–1.13) |
| *P* for trend | | 0.63 | | 0.16 | |
| Cycling | | | | | |
| No | 1408/1511 | 1.00 | – | 1.00 | – |
| Yes | 46/37 | 1.33 | (0.86–2.07) | 1.38 | (0.86–2.20) |
| *P* Chi-square | | 0.20 | | 0.18 | |
| *Adult (last 10 yrs)* | | | | | |
| Household activity (h $wk^{-1}$) | | | | | |
| 0–1 | 195/204 | 1.00 | – | 1.00 | – |
| 2 | 358/353 | 1.03 | (0.81–1.32) | 1.06 | (0.82–1.38) |
| 3 | 404/453 | 0.91 | (0.71–1.15) | 0.96 | (0.74–1.24) |
| 4 | 326/342 | 0.95 | (0.74–1.23) | 1.03 | (0.79–1.34) |
| ≥ 5 | 176/202 | 0.84 | (0.63–1.12) | 0.90 | (0.66–1.23) |
| *P* for trend | | 0.15 | | 0.45 | |
| Walking (min $d^{-1}$) | | | | | |
| 0–14 | 245/281 | 1.00 | – | 1.00 | – |
| 15–29 | 143/161 | 0.99 | (0.75–1.31) | 1.02 | (0.76–1.38) |
| 30–59 | 400/392 | 1.13 | (0.90–1.41) | 1.18 | (0.93–1.48) |
| 60–79 | 386/397 | 1.05 | (0.84–1.31) | 1.13 | (0.89–1.42) |
| ≥80 | 282/324 | 0.92 | (0.73–1.18) | 1.00 | (0.78–1.28) |
| *P* for trend | | 0.69 | | 0.79 | |
| Cycling (min $d^{-1}$) | | | | | |
| Zero | 1029/1074 | 1.00 | – | 1.00 | – |
| 1–29 | 74/83 | 0.98 | (0.71–1.36) | 1.02 | (0.73–1.43) |
| 30–39 | 100/93 | 1.19 | (0.88–1.61) | 1.18 | (0.87–1.60) |
| 40–59 | 71/90 | 0.88 | (0.63–1.22) | 0.85 | (0.60–1.19) |
| ≥60 | 183/207 | 0.98 | (0.79–1.22) | 0.97 | (0.77–1.22) |
| | | 0.78 | | 0.68 | |

[1]Adjusted for age, education, household income, first-degree family history of breast cancer, history of breast fibroadenoma, age at menarche, age at first live birth, and age at menopause.
[2]Numbers of cases and controls vary due to non-reporting on some variables.

**Table 6** Odds ratios[1] (OR) and 95% confidence limits (95% CL) for breast cancer associated with lifetime exercise activity by quintile of lifetime occupational activity, the Shanghai Breast Cancer Study (1996–1998)

| | Quintiles of occupational standing or walking (h $d^{-1}$) | | | | | | | | | |
|---|---|---|---|---|---|---|---|---|---|---|
| | **0.0–0.9 (*n* = 622)** | | **1.0–2.3 (*n* = 619)** | | **2.4–3.9 (*n* = 553)** | | **4.0–5.9 (*n* = 631)** | | **≥6.0 (*n* = 598)** | |
| Adolescent/Adult | | | | | | | | | | |
| No/No | 1.00[2] | – | 0.69 | (0.50–0.95) | 0.64 | (0.45–0.92) | 0.77 | (0.55–1.06) | 0.54 | (0.39–0.76) |
| Yes/No | 0.70 | (0.47–1.05) | 0.60 | (0.41–0.89) | 0.52 | (0.34–0.80) | 0.65 | (0.44–0.95) | 0.55 | (0.37–0.82) |
| No/Yes | 0.84 | (0.50–1.41) | 0.94 | (0.53–1.65) | 0.24 | (0.12–0.49) | 0.52 | (0.31–0.89) | 0.30 | (0.17–0.54) |
| Yes/Yes | 0.42 | (0.24–0.73) | 0.44 | (0.25–0.78) | 0.21 | (0.10–0.44) | 0.41 | (0.23–0.75) | 0.23 | (0.12–0.46) |
| *P* for trend | <0.01 | | 0.01 | | <0.01 | | <0.01 | | <0.01 | |

[1]Adjusted for age, education, household income, first-degree family history of breast cancer, history of breast fibroadenoma, age at menarche, age at first live birth and age at menopause. [2]Referent category for all comparisons are No/No exercisers and the lower quintile of occupational activity (0.0–0.9 h $d^{-1}$).

*© 2001 Cancer Research Campaign*

et al, 1998), reducing oestrogen exposure early in the follicular phase and progesterone exposure in the luteal phase (De Souza et al, 1998), and by shortening the luteal phase (Loucks et al, 1989; De Souza et al, 1998), a time of increased cell proliferation in the breast (Pike et al, 1993). Recent reports suggest additional biologic mechanisms for reducing breast cancer risk via the insulin-like growth factor-I (IGF-1) in younger women. High levels of regular physical activity would be expected to minimize the development of insulin resistance and hyperinsulinaemia (Mayer-Davis et al, 1998), which would be predicted to reduce biologically active IGF-I levels, and therefore breast cancer risk (Kaaks, 1996; Stoll, 1999).

Consistently high physical activity levels in adolescence and throughout adulthood would minimize adult weight gain and the accumulation of visceral adipose stores (Kohrt et al, 1992a, 1992b), both of which have been associated with increased post-menopausal breast cancer risk (Ballard-Barbash, 1994; Huang et al, 1997). The increase in risk with postmenopausal adiposity appears to be driven by its effect on biologically available oestrogen levels (Toniolo et al, 1995). Physical activity also has been noted to be independently associated with lower oestrone levels in postmenopausal women (Cauley et al, 1989). Taken together, there are several plausible biologic mechanisms for a reduced risk of breast cancer both pre- and post-menopausally.

Case–control studies are subject to selection and information biases. Selection bias in this study is unlikely to give the comparable participation rates for both cases (91%) and controls (90%). We observed little or no confounding in our primary analyses, even after controlling for most known breast cancer risk factors. It is conceivable that certain dietary factors may act as a confounder here but given the strength of the associations reported here confounding seems unlikely to fully explain the associations reported. In unpublished analyses we have found no mean differences between cases and controls for energy intake (1866 (464) vs. 1840 (464) kcal $d^{-1}$, $P = 0.12$) and fat intake (36.3 (17.4) vs. 35.3 (16.2) g $d^{-1}$, $P = 0.08$) (values are cases vs. control, mean (SD)). Differential recall biases by disease status are possible, but are less likely in contexts with little emphasis on the putative role of physical activity in breast cancer. Were activity to be severely limited by the presence of disease, another kind of recall bias could be introduced namely, the conditioning of memory by current experience and expectation. However, most of the women in this study would not have had limited physical capability as a consequence of their disease.

Interviewer-derived assessments of lifetime physical activity have been observed to be reproducible (Friedenreich et al, 1998a) and to provide reasonable recall accuracy for periods of 2 (Slattery and Jacobs, 1995) to 10 years (Blair et al, 1991). Reporting errors in historical activity patterns would appear to be more problematic for the quantitative aspects (i.e., frequency, intensity, duration of activity), but less so for the dichotomous assessment of exercise (i.e., yes/no) as used in the present study. There is little information on the validity of historical occupational activity recall. However, the high level of structure and repetition of activity in many occupations, repeated over many years of a person's life, should enhance recall, suggesting the feasibility of differentiating sedentary from high-activity occupations. Our finding of an inverse association only with our most individualized quantitative assessment of occupational activity (i.e., h $d^{-1}$ of standing or walking) underlines the limitations of less specific assessments, using relative self-ratings of physical activity or occupational classifications based on job codes. It was notable that our measures of daily activities (i.e., h $d^{-1}$ of household activity, cycling and walking) that did not incorporate information about the years of participation in a particular life-period failed to show an effect. This suggests an important element in the assessment of historical activity patterns is the length of physical activity exposure.

Amelioration of the physical activity-breast cancer relationship in overweight women has been observed in only 2 of 6 published studies (Friedenreich et al, 1998b), and few of these evaluated pre- and post-menopausal women separately. Such evaluation would seem important given the differential effect of obesity on breast cancer risk (Ballard-Barbash, 1994; Bernstein and Ross, 1993). We observed little or no modification of the physical activity-breast cancer association by overweight status (i.e., BMI $< 25$ vs. BMI $\geq 25$ kg/$m^2$) in pre- or post-menopausal women.

In conclusion, our findings add support to the notion that high levels of physical activity throughout life reduce the risk of breast cancer. They also suggest that initiating exercise in adulthood may reduce breast cancer risk by 30%, a finding potentially of considerable public health importance.

### ACKNOWLEDGEMENTS

This research was supported by USPHS Grant RO1CA64277 to Dr. Wei Zheng.

### REFERENCES

AICR (1997) Cancers, nutrition, and food: breast. In: *Food, Nutrition and the Prevention of Cancer: a global perspective* pp. 252–287. American Institute for Cancer Research: Washington, DC

Ainsworth B, Haskell W, Whitt M, Irwin M, Swartz A, Strath S, O'Brien W, Bassett D, Schmitz K, Emplaincourt P, Jacobs D and Leon A (2000) Compendium of physical activities: an update of activity codes and MET intensities. *Med Sci Sports Exerc* **32**: S498–S516

Ballard-Barbash R (1994) Anthropometry and breast cancer. Body size – a moving target. *Cancer* **74**: 1090–1100

Bernstein L and Ross RK (1993) Endogenous hormones and breast cancer risk. *Epidemiol Rev* **15**: 48–65

Bernstein L, Ross RK, Lobo RA, Hanisch R, Krailo MD and Henderson BE (1987) The effects of moderate physical activity on menstrual cycle patterns in adolescence: implications for breast cancer prevention. *Br J Cancer* **55**: 681–685

Bernstein L, Henderson BE, Hanisch R, Sullivan-Halley J and Ross RK (1994) Physical exercise and reduced risk of breast cancer in young women. *J Natl Cancer Inst* **86**: 1403–1408

Blair S, Dowda M, Pate R, Kronenfeld J, Howe H, Parker G, Blair A and Fridingner F (1991) Reliability of long-term recall of participation in physical activity by middle-aged men and women. *Am J Epidemiol* **133**: 266–275

Carpenter CL, Ross RK, Paganini-Hill A and Bernstein L (1999) Lifetime exercise activity and breast cancer risk among post-menopausal women. *Br J Cancer* **80**: 1852–1858

Cauley JA, Gutai JP, Kuller LH, LeDonne D and Powell JG (1989) The epidemiology of serum sex hormones in postmenopausal women. *Am J Epidemiol* **129**: 1120–1131

De Souza MJ, Miller BE, Loucks AB, Luciano AA, Pescatello LS, Campbell CG and Lasley BL (1998) High frequency of luteal phase deficiency and anovulation in recreational women runners: blunted elevation in follicle-stimulating hormone observed during luteal-follicular transition. *J Clin Endocrinol Metab* **83**: 4220–4232

Friedenreich CM, Courneya KS and Bryant HE (1998a) The lifetime total physical activity questionnaire: development and reliability. *Med Sci Sports Exerc* **30**: 266–274

Friedenreich CM, Thune I, Brinton LA and Albanes D (1998b) Epidemiologic issues related to the association between physical activity and breast cancer. *Cancer* **83**: 600–610

Friedenreich CM, Bryant HE and Courneya KS (2001) Case-control study of lifetime total physical activity and breast cancer risk. *Am J Epidemiol* in review

 © 2001 Cancer Research Campaign

Frisch RE, Wyshak G, Albright NL, Albright TE, Schiff I, Jones KP, Witschi J, Shiang E, Koff E and Marguglio M (1985) Lower prevalence of breast cancer and cancers of the reproductive system among former college athletes compared to non-athletes. *Br J Cancer* **52**: 885–891

Gao YT, Shu XO, Dai S, Potter JD, Brinton LA, Wen W, Sellars TA, Kushi LH, Ruan Z, Bostick RM, Jin F and Zheng W (2000) Association of menstrual and reproductive factors with breast cancer risk: results from the Shanghai Breast Cancer Study. *Int J Cancer* (in press)

Hosmer DW and Lemeshow S (1989) *Applied Logistic Regression*. John Wiley & Sons: New York

Huang Z, Hankinson SE, Colditz GA, Stampfer MJ, Hunter DJ, Manson JE, Hennekens CH, Rosner B, Speizer FE and Willett WC (1997) Dual effects of weight and weight gain on breast cancer risk. *JAMA* **278**: 1407–1411

Kaaks R (1996) Nutrition, hormones, and breast cancer: is insulin the missing link? *Cancer Causes Contr* **7**: 605–625

Kohrt WM, Malley MT, Dalsky GP and Holloszy JO (1992a) Body composition of healthy sedentary and trained, young and older men and women. *Med Sci Sports Exerc* **24**: 832–837

Kohrt WM, Obert KA and Holloszy JO (1992b) Exercise training improves fat distribution patterns in 60- to 70-year-old men and women. *J Gerontol* **47**: M99–105

Loucks AB, Mortola JF, Girton L and Yen SS (1989) Alterations in the hypothalamic-pituitary-ovarian and the hypothalamic-pituitary-adrenal axes in athletic women. *J Clin Endocrinol Metab* **68**: 402–411

Mayer-Davis EJ, D’Agostino R, Karter AJ, Haffner SM, Rewers MJ, Saad M and Bergman RN (1998). Intensity and amount of physical activity in relation to insulin sensitivity: The Insulin Resistance Atherosclerosis Study. *JAMA* **279**: 669–674

Merzenich H, Boeing H and Wahrendorf J (1993) Dietary fat and sports activity as determinants for age at menarche. *Am J Epidemiol* **138**: 217–224

Pike MC, Spicer DV, Dahmoush L and Press MF (1993) Estrogens, progestogens, normal breast cell proliferation, and breast cancer risk. *Epidemiol Rev* **15**: 17–35

Rockhill B, Willett WC, Hunter DJ, Manson JE, Hankinson SE and Colditz GA (1999) A prospective study of recreational physical activity and breast cancer risk. *Arch Int Med* **159**: 2290–2296

Sellers TA (1997) Genetic factors in the pathogenesis of breast cancer: their role and relative importance. *Journal of Nutrition* **127**: 929S–932S

Slattery ML and Jacobs DR, Jr (1995) Assessment of ability to recall physical activity of several years ago. *Ann Epidemiol* **5**: 292–296

Stoll BA (1999) Western nutrition and the insulin resistance syndrome: a link to breast cancer. *Eur J Clin Nutr* **53**: 83–87

Thune I, Brenn T, Lund E and Gaard M (1997) Physical activity and the risk of breast cancer: *N Engl J Med* **336**: 1269–1275

Toniolo PG, Levitz M, Zeleniuch-Jacquotte A, Banerjee S, Koenig KL, Shore RE, Strax P and Pasternack BS (1995) A prospective study of endogenous estrogens and breast cancer in postmenopausal women. *J Natl Cancer Inst* **87**: 190–197

Verloop J, Rookus MA, van der Kooy K and van Leeuwen FE (2000) Physical activity and breast cancer risk in women aged 20–54 Years. *J Natl Cancer Inst* **92**: 128–135

Walker RA, Lees E, Webb MB and Dearing SJ (1996) Breast carcinomas occurring in young women (<35 years) are different. *Br J Cancer* **74**: 1796–1800

Wyshak G and Frisch RE (2000) Breast cancer among former college athletes compared to non-athletes: a 15-year follow-up. *Br J Cancer* **82**: 726–730

*© 2001 Cancer Research Campaign*
